# Data Fusion of Electronic Nose and Multispectral Imaging for Meat Spoilage Detection Using Machine Learning Techniques

**DOI:** 10.3390/s25103198

**Published:** 2025-05-19

**Authors:** Vassilis S. Kodogiannis, Abeer Alshejari

**Affiliations:** 1 College of Design, Creative and Digital Industries, University of Westminster, London W1W 6UW, UK; 2 Department of Mathematical Science, Princess Nourah bint Abdulrahman University, Riyadh 11671, Saudi Arabia; aaalshejari@pnu.edu.sa

**Keywords:** neural networks, fuzzy logic, meat spoilage, feature selection, multispectral imaging, electronic nose, machine learning

## Abstract

Meat quality plays a significant role in the consumers’ health condition; hence, the constant pursuit for techniques capable of objective and accurate quality assessment by the meat industry. Multispectral imaging and electronic noses are valuable techniques for the rapid and non-destructive detection of meat spoilage. In order to take advantage of the complementary information provided by these two different sensing devices, a high-level data fusion strategy was explored. Through this fusion scheme, the aim of this work is to estimate initially the population of total viable counts of *Pseudomonas* spp., *Brochothrix thermosphacta* and lactic acid bacteria, and then to categorize the status of the meat samples into three classes (fresh, semi-fresh, and spoiled). The issue of small size available datasets was addressed by generating additional “virtual” sample sets, through the use of neural networks. Neuro-fuzzy based regression models were implemented and their outputs were combined in order to estimate these microbiological populations. Following the evaluation of these estimations, it can be argued that the most efficient prediction was obtained through the fusion of these sensing devices, the coefficients of determination, the residual prediction deviation, and the range error ratio exceeded the 0.98%, 5.4%, and 14.73%, respectively. In parallel, the classification rate for the grouping of the testing samples into three classes was perfect. Based on the acquired results, the proposed analytical concept could potentially provide an alternative approach towards the efficient detection of meat spoilage.

## 1. Introduction

One of the main concerns for the food industry is related to the quality and cost of their products, as consumers always take into consideration these factors [1]. Thus, food quality and safety levels have always been a central issue for discussion, as well as for taking appropriate actions to address them. The consumption of meat, which is generally considered to be an essential part of our diet, is due to the fact that it is rich in protein and contains high physiological value. This type of consumption, which includes pork, beef, and poultry, is increasing worldwide every year, and based on the 2017 report from the Organization for Economic Co-operation and Development (OECD), it was predicted that the average meat consumption per person could approach to 35.5 kg globally by 2024 [2]. A market survey has indicated that, alongside the growth of meat consumption, meat quality is gradually becoming an essential issue in the consumers’ purchasing decisions [3]. Despite the fact that meat is considered to be a good source of protein and other essential nutrients, it is also a suitable environment for the growth and survival of spoilage and pathogenic microorganisms. Spoilage occurs when the formation of off-flavors, off-odors, discoloration, or any other changes in physical appearance or chemical characteristics make the food unacceptable to the consumer. Changes in the muscle characteristics are due to native or microbial enzymatic activity or to other chemical reactions. However, not all bacteria are responsible for the spoilage effect; there is only an initial small group of microorganisms in meat, named as specific spoilage organisms (SSOs) [4]. In meat products, SSOs metabolize the available substrates during storage, thus leading to changes in the meat quality and odor. The current procedure for checking the levels of meat spoilage is performed either subjectively, based on a sensory assessment, or through a microbiological analysis. Such sensory assessments usually utilize the human senses of a trained test panel to provide evidence related to color, smell, and taste, as well as the overall quality and acceptance of the meat sample. This approach, though widely utilized for the classification of meat samples, has some weaknesses, such as the high cost for training the taste panel, the reproducibility of the evaluation, and the potential low comparability between panels [5].

Alternatively, the conventional microbiological approach to food sampling has changed little over the previous decades and it is based on the recording of bacterial counts for a given sample as a quantitative indicator of spoilage. These bacterial counts include the total viable count (TVC), *Pseudomonas* spp., *Brochothrix thermosphacta*, *Enterobacteriaceae*, and lactic acid bacteria. The “standard plate count” approach was used as the main microbiological method where the sample, after the preparation and dilution stages, is mixed with a general agar media, incubated, and then the colonies are counted after 48 h. Despite its simplicity, it is a time-consuming process which employs an enormous amount of culture media, a large number of sterile test materials, as well as large incubation spaces. Biosensors represent one advanced method that was developed to provide faster microbiological information compared to the conventional microbiological approach. In the field of microorganism detection, adenosine triphosphate (ATP) bioluminescence, an effective biosensor, acts by measuring the ATP levels in bacterial cells in a culture in order to calculate the number of cells present. Although the detection time is about one to four hours, the problem with this method is that the ATP present in the meat has to be destroyed before the microbial ATP can be measured [6]. Alternatively, a polymerase chain reaction (PCR) has successfully been used to detect microorganisms by amplification of the target DNA and detecting the target PCR products. This specific type of nucleic acid-based detection method requires the presence of intact nucleic acid sequences in the sample. Thus, the DNA from non-viable microorganisms can lead to false positive results. Another limitation is the time factor, as this can be a time-consuming method compared to ATP [7].

While some of these methods are superior to others, and most of them provide adequate results, their main drawback, at present, is the time taken to acquire results. The optimal solution for the food industry would be a rapid, non-destructive, reagent-less, quantitative, and relatively inexpensive method for microbiological analysis. Thus, inexpensive, fast, and non-invasive methods have been explored for this purpose: to provide an alternative and reliable solution for meat spoilage detection. Such methods include various analytical lab instruments, like Fourier transform infrared spectroscopy (FTIR) [8], hyperspectral and multispectral imaging systems [9], Raman spectroscopy [10], and electronic noses (e-nose) [11]. The detection “capability” of these analytical instruments is based on the hypothesis that any produced metabolic activity from each meat sample is considered to be an individual “signature”, which practically contains important information for the level of quantitative indicators responsible for spoilage [12]. However, the main issue with these new techniques is how to associate their produced output with the indicators responsible for spoilage, as well as with the output of a sensory assessment for the overall quality and acceptance of the meat sample. Fortunately, with the advancement of computing software, algorithmic models have been trained (offline) to associate sensorial outputs with meat quality indicators, and then the final developed models can be utilized as rapid decision models without the need of additional microbiological tests.

Due to the complexity of the chemical-based characteristics that appear in food products, the application of a single analytical instrument/sensor may not be sufficient, and multi-sensor data fusion techniques, combining the outputs of multiple instruments/sensors, could provide an alternative challenge for improving the level of the assessment of food quality [13].

Data fusion is an emerging branch in chemometrics that analyzes the combination of information provided by different instruments, since various sources of data can potentially provide complementary information compared with the case of a single data source. Three different fusion strategies have been designed, commonly named low-level data fusion (LLF), mid-level feature fusion (MLF), and high-level decision fusion (HLF) [14]. The LLF involves the collection of data from different sensors for the same samples, which are then directly concatenated into a single matrix (after proper pre-processing) to obtain a new, larger dataset. The limitations of LLF include the presence of a high volume of data and the possible predominance of one data source over the others. Unlike LLF, the MLF (feature level fusion) strategy integrates a feature extraction step which can incorporate adequate original information, with the extracted features combined to build quantitative or qualitative models. Previous issues encountered by the LLF are somehow resolved in the MLF, as extraction significantly reduces the data dimensionality. In this scheme, feature selection techniques and principal component analysis (PCA) are widely employed. Feature level fusion is very useful for non-commensurate type data, i.e., if sensors are looking for different physical parameters. However, the real challenge in this strategy is to find the optimal combination of extracted features and pre-processing that describes the significant variation of the original sensorial responses and provide the best final model. In the HLF (decision level fusion), models are separately developed for each individual sensor and the respective results are then integrated into a single final response. One advantage of this scheme is that each individual produced model is treated independently; as such, inferior performance from one model does not worsen the overall performance, unlike the other fusion strategies. The challenge with this scheme, however, is that special care is required to determine the most accurate individual models so that the combination of their outputs will produce a superior performance.

Several approaches of the data fusion methods have been employed for meat quality monitoring in terms of discrimination, adulteration, and prediction. Such case studies have led to an interest in exploring data fusion methodologies that could decrease the uncertainty of individual results and enable a superior performance in prediction. In one study, a decision fusion method based on hyperspectral imaging (HSI) and an electronic nose (e=nose) technique for moisture content prediction in frozen-thawed pork was explored by comparing various approaches to extract the required features, while a partial least squares (PLSR) regression model provided the prediction for moisture [15]. In another study, the prediction of two important indicators, namely the total volatile basic nitrogen (TVB-N) and TVC, for evaluating the quality of chicken fillets was investigated through the use of two different HSI techniques, visible near-infrared (Vis-NIR) and NIR. Quantitative predictions using PLSR were calculated after the feature wavelength selection [16]. A low-cost e-nose was fused with Fourier transform-near-infrared (FT-NIR) spectroscopy to detect the level of beef adulteration with duck. The TVB-N, protein, fat, total sugar, and ash contents were measured to investigate the differences in basic properties between the raw beef and the duck, while extreme learning based machine models were developed to identify the adulterated beef and predict the adulteration levels [17]. Robert et al. explored the fusion of different spectroscopic techniques for meat analysis. Mid infrared (MIR), near infrared (NIR), and Raman spectroscopy were fused in an LLF scheme to estimate fatty acid composition in processed lamb using PLSR models [18]. In a prior study, Robert et al. investigated the performance of LLF, MLF, and HLF schemes of Raman and infrared spectroscopy to predict pH and the percentage of intramuscular fat content (% IMF) for red meat quality parameters utilizing PLSR models. The HLF approach proved able to provide the best performance for the pH parameter, while the LLF showed promising results in predicting the percentage of IMF quality [19].

The main objective of this paper is to detect beef spoilage during aerobic storage at various temperatures (0, 4, 8, 12, and 16 °C) through an advanced intelligence-based decision support system. An HLF strategy of spectral information acquired by a multispectral imaging (MSI) system and volatile fingerprints of odor profile obtained through an e-nose will be utilized as a basis for the development of the proposed decision system. The proposed analytical framework aims not only to predict the levels of meat indicators (total viable counts, *Pseudomonas* spp., *Brochothrix thermosphacta*, and lactic acid bacteria) encountered in beef samples but to categorize beef samples into three distinct classes (i.e., fresh, semi-fresh, and spoiled).

Data quantity is generally an issue of concern for machine learning applications, as small datasets usually do not lead to a robust classification/prediction performance. How to create some additional information from a small dataset is thus of considerable interest. In the proposed fusion of MSI and e-nose devices, unfortunately, the individual obtained sensorial data are not only limited but not equal in terms of the number of samples. This latter issue practically introduces an inconsistency in fusing the information acquired by different types of sensors. In this research, an efficient methodology for creating additional “virtual” sample sets, thus improving the accuracy of the proposed decision support system, was proposed. Inspired by the way the radial basis function (RBF) neural network manages to approximate levels of microorganisms [20], a forward modeling process was used to create additional “virtual” outputs for the levels of the meat indicators that need to be predicted. In addition, an inverse RBF-based modeling process was used to create additional “virtual” sensorial outputs for both the MSI and e-nose systems. The enhanced datasets for both instruments, which include the additional “virtual” information, are then subjected to a feature selection analysis, based on the Boruta algorithm, to identify the most important features for both sensors. The selected features are then utilized as inputs to regression models built to approximate relevant meat indicators for each sensorial device. The related models’ outputs are then combined to provide the overall prediction. Finally, based in these final predictions, a simple implemented classifier predicts the class of meat samples also utilizing information from the provided sensory assessment. As the “heart” of the proposed analysis is related to the development of accurate regression models for each meat indicator, an adaptive fuzzy logic neural system (AFLS) was employed for this task. Testing performances of the AFLS models are compared against the models usually employed to related food microbiological applications, such as the PLSR and the multilayer perceptron (MLP), as well as against traditional machine learning models, such as support vector machines (SVM) and extreme gradient boosting (XGBoost), using a number of established evaluation metrics. The overall “idea” of the implemented methodology is to highlight the concept of multi-fusion analysis using advanced learning-based models in the area of food microbiology.

## 2. Experimental Case

### 2.1. Sample Preparation and Microbiological Analysis

The whole experimental work, as well as the related information acquired from the application of the MSI and e-nose instruments to beef samples, was performed at the Agricultural University of Athens, Greece. All detailed information related to the microbiological as well as the sensorial analysis of the beef samples used in these tests can be found in [5,21]. In brief, small pieces of fresh beef fillets were stored at the following temperatures 0, 4, 8, 12, and 16 °C, under aerobic conditions, in incubators for up to 434 h, subjected to storage temperature, until the spoilage effect was identified. This limited range of temperatures was chosen to reflect adequately the monitoring of the spoilage effect, also taking into consideration the practical limitations in such type of experiments. Due to time and cost constraints, generally it is not possible to collect a wide range of real samples in the area of food analysis, thus a suitable data analysis can assist researchers to overcome this practical limitation. Duplicate samples were then collected for these storage conditions at specific distinct time-steps in order to be utilized for the microbiological as well as the sensorial-based analysis.

For the microbiological analysis, the following media were utilized to calculate specific meat indicators. Total viable counts were enumerated on plate count agar (PCA), while *Pseudomonas* spp. and *Brochothrix thermosphacta* were cultivated on a *Pseudomonas* CFC selective agar (CFC) and a streptomycin thallous acetate actidione (STAA) agar medium, respectively. Finally, for the case of lactic acid bacteria, enumeration was performed via Man–Rogosa–Sharpe (MRS) agar. The acquired growth data were log_10_ transformed and, through a model developed by Baranyi, specific kinetic parameters of microbial growth (maximum growth rate as well as lag phase length) for the estimation of these four meat indicators were calculated [21]. For this research study, although 84 beef samples were used in microbiological analysis as well as in the MSI-based experiment, only a subset, comprising 58 samples, was utilized in the e-nose experiment. This inconsistency in the number of real samples utilized in these two sensorial-based experiments has been addressed through the creation of additional synthetic data in order to maintain a uniformity during the fusion data analysis.

**Figure 1:**
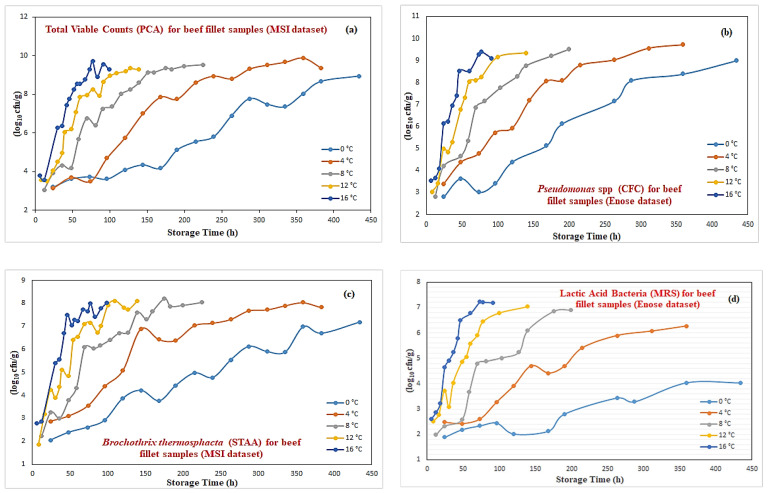
Growth curves for total viable counts (**a**), *Pseudomonas* spp. (**b**), *Brochothrix thermosphacta* (**c**), and lactic acid bacteria (**d**) at various temperatures.

The growth curves for the total viable counts (PCA agar), *Pseudomonas* spp. (CFC agar), *Brochothrix thermosphacta* (STAA agar), and lactic acid bacteria (MRS agar) for the obtained beef samples at these specific temperatures as a function of storage time are illustrated at Figure 1a–d. An inspection at these graphs revealed that the growth rate of the total viable counts graph increased faster as the storage temperature increased. This is in agreement with the concept that any potential increase in temperature also affects the number of bacteria responsible for spoilage. The maximum specific growth rate of *Pseudomonas* seems to be comparable to the total viable counts, but also higher than of that of the other two remaining microorganisms, with *Brochothrix thermosphacta* following very closely. Finally, although the growth rate for lactic acid bacteria was always below the others, such difference is diminished as the storage temperature increases.

### 2.2. Sensory Assessment

In parallel to the microbiological analysis, a sensory evaluation of the beef samples was performed by a sensory panel at the same time intervals also used in the microbiological analysis [5,21]. The evaluation was performed in artificial light, and all samples were left to reach ambient temperature before starting the assessment where samples were scored based on the perception of color, smell, and taste. The color and odor were determined before and after cooking (20 min at 180 °C in a preheated oven), whereas taste was defined after cooking.

A three-class evaluation scheme was performed with one class to be associated with the beef samples, that are characterized by the absence of off-flavors and are suitable for consumption (fresh). Bright colors typical of fresh oxygenated meat were considered to be an indication of fresh meat. Another class that corresponded to clearly off-flavor development for which the sample was of an unacceptable quality was characterized as spoiled. In this category, putrid, sweet, sour, or cheesy odors were considered to be indicators of possible microbial spoilage. Finally, the remaining samples were categorized as semi-fresh. For this group, an indication of change from that of typical fresh meat (i.e., less bright red color, odor and flavor slightly changed, but still acceptable quality) was observed.

Table 1 provides a summary of growth ranges for all meat indicators (shown with their agar medium used) and their association with the groupings provided by sensory assessment. These results again indicate the direct relation of temperature and bacteria growth ranges. In most cases, a clear separation between the classes can be shown, although in some cases a small overlapping of growth ranges between classes can be observed. This can be explained by the fact that microbiological and sensory assessments were performed independently and potential errors, either by the sensory panel or by the computational model used to extract the growth ranges, can be found. Nevertheless, the information included in Table 1 is considered to be valuable, as the main objective of this research is the prediction of growth for each meat indicator, as well as the prediction of class through the use of an intelligent model which will utilize sensorial outputs and temperature/time parameters as model descriptors.

### 2.3. Electronic Nose Acquisition

The acquired volatile fingerprints of odor profile from each meat sample were obtained through the use of the Libra e-nose [5]. The instrument, which was produced by Technobiochip (Elba Island, Italy) [22], is composed by an array of eight 20 MHz quartz crystal microbalance (QCM) transducers/sensors coated with different poly-pyrrole polymer films and deposited by the Langmuir–Schaefer technique via a KSV 5000 instrument (KSV Instruments, Helsinki, Finland). These polymer films were produced by the reaction of pyrrole with different compounds (i.e., aldehydes); Table 2 summarizes the aldehydes used for the chemical synthesis and the poly-pyrrole derivatives obtained.

**Figure 2:**
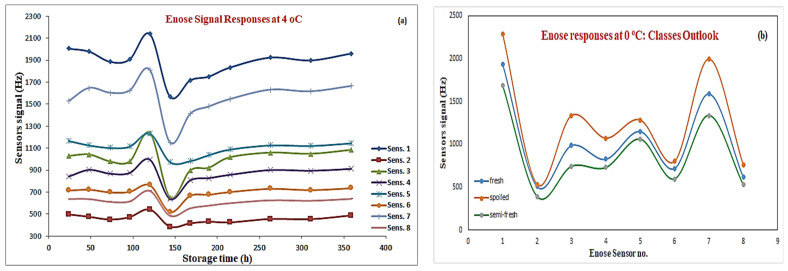
Illustration of “volatile” responses at 4 °C (**a**) & responses based on different classes (**b**).

These piezoelectric, in nature, transducers were placed in a measuring chamber. The measuring chamber was held at a constant temperature during the measurements by a thermostatic electronic system, while a flow system formed by a micro-electric valve and a micro-pump transmits the gas sample to the measuring chamber. The functionality of each sensor is based on the mass variation (Δm) of the quartz surface, due to a direct interaction between the sensing element and the volatile compounds. Based on a relation, known as the Sauerbrey Law, the frequency–mass relationship can be defined, thus the maximum increment of frequency between the initial and final frequency, after the sensor’s exposure to the gas sample (Δfmax), can be obtained and used as the sensor’s response for that specific volatile compound [23]. These sensors operate like biological receptors, and the integrated data analysis system allows us to transpose information that the sensors extract from an odor in an “olfactory image” analogous with our “sensation” of a smell. The detection of odors is based on the perception that different odors are associated with different “olfactory” images. This characteristic differentiates an e-nose from gas chromatography where single molecular types inside a gaseous mixture are identified. In the e-nose case, an odor is recognized as a whole, showing thus the synergic activity of different molecular species into a single “olfactory” image. For each measurement, a small beef fillet sample was introduced inside a 100 mL volume glass jar and left at room temperature (20 ± 2 °C) for approx. 15 min (including the necessary cleaning of the sensors in order to perform a single measurement) to enhance desorption of volatile compounds from the sample into the headspace. The headspace was then pumped over the e-nose sensors and the generated signal was recorded by a computer. Data related to the volatile extracted information from the e-nose was further utilized towards the development of the proposed intelligent-based analytical framework. The responses of all sensor signal classes for meat samples stored at 4 °C are shown in Figure 2. Based on the three-class evaluation scheme by the evaluation taste panel, the acquired 58 beef samples processed through the e-nose based experiment were classified as fresh (15 samples), semi-fresh (19 samples), and finally spoiled (24 samples).

The e-nose signal responses, shown in Figure 2a, reveal a “rapid” slope characteristic which can be seen in the middle of the graph. The area of this slope is related to semi-fresh samples, at 4 °C, and a storage time between 73 and 144 h is “allocated” by the sensory assessment to samples belonging to that specific category. Figure 2b illustrates the responses of the e-nose sensors to samples belonging to different classes for samples stored at 0 °C.

### 2.4. Multispectral Imaging Acquisition

Images of the beef fillets were captured through the use of VideometerLab (Videometer A/S, Hørsholm, Denmark), which acquired multispectral images at 18 distinct wavelengths ranging from visible to the NIR region, and the values of the measured wavelengths were 405, 435, 450, 470, 505, 525, 570, 590, 630, 645, 660, 700, 850, 870, 890, 910, 940, and 970 nm. Each meat sample, presented in a Petri dish, was set in a specially made (Ulbricht) sphere, which was coated with matte titanium paint to ensure a uniform reflection of the projected light so that a uniform light formed on the entire sphere. A monochrome CCD imaging sensor on top of the sphere gathered the surface reflections and recorded the data. Light-emitting diodes (LEDs) were set at the rim of the sphere and each wavelength of LEDs with narrowband spectral radiation distribution was evenly distributed across the entire edge. The result was a monochrome image with 32-bit floating point precision for each LED type, providing, at the end, a multispectral 3D cube of dimensionality 1280 × 960 × 18 [24].

The associated VideometerLab (version 2.12.39) software was used to perform imaging tasks, such as image segmentation, into discrete regions. Following the segmentation process for each specific wavelength, the mean reflectance spectrum was calculated by averaging the pixels’ intensity in the region of interest (ROI). The acquired mean reflectance values (totally 18 attributes) for each meat sample practically constitute the “spectral signature” for that specific sample.

**Figure 3:**
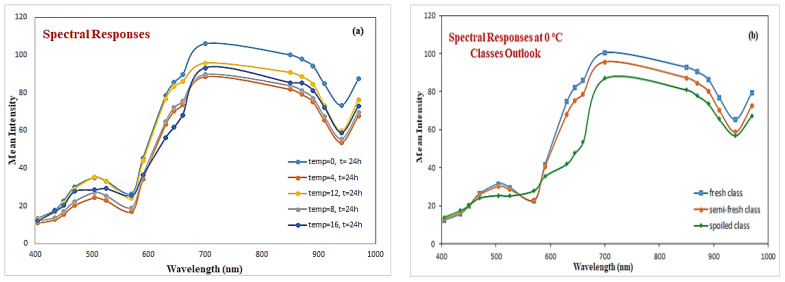
Illustration of selected MSI-based spectral responses (**a**) & spectral responses based on different classes (**b**).

Figure 3a illustrates an example of the mean reflectance spectra acquired from the beef fillet samples at various temperatures at the same time step (i.e., 24 h). It is interesting to note that these specific samples have been categorized, by the sensory assessment, as fresh samples, with the exception of the sample with temp = 16, which was classified as semi-fresh. The schematic shown in Figure 3a reveals not only the complex dynamic systems that characterize these biological cases (i.e., the prediction of meat indicators) but the influence of time and temperature parameters to the behavior of such cases. Figure 3b illustrates the spectral responses of beef samples stored under the same temperature but collected at different time-steps, thus providing an indication of the applicability of the specific sensorial method to detect meat spoilage.

A close look at these selected “mean” spectra in Figure 3a lead to some interesting conclusions. Although these selected samples are associated with different temperatures, their “mean” spectra follow a common pattern, where an increased trend in the reflectance’s magnitude, especially for the visible part (400–700 nm), can be noticed, while their related reflectance values are decreased in the near infrared range. According to the literature, most of the spectral information used for meat discrimination is contained in the visible and near infrared region [25], therefore the adopted feature selection process using the Boruta method will try to verify this specific scenario. Following the sensory assessment review, the 84 meat spectral samples obtained through the MSI-based experiment were categorized as fresh (16 samples), semi-fresh (20 samples), and finally spoiled (48 samples).

## 3. Synthetic Data Acquisition and Proposed Analytical Framework

The availability of data in terms of high quality/quantity is essential for the success of various applications across a wide range of fields. Large datasets are needed because of the basic idea that insights from such datasets may adjust decision-making and reveal previously unnoticed patterns. Unfortunately, small dataset conditions exist in many fields, such as food analysis, disease diagnosis, fault diagnosis, or deficiency detection in mechanics, among others. In many cases/applications, it is not possible to obtain a large amount of information, due to a number of reasonable causes. The main reason that small datasets cannot provide adequate information, unlike large datasets, is that gaps between the samples may exist; even the domain of the samples cannot be ensured [26]. Thus, it is difficult with a small dataset to approximate the pattern of high order nonlinear functions through a standard machine learning model, since small sets have shown weakness in providing the necessary information for forming population patterns. Hence, for a learning system that lacks sufficient data, the knowledge learned is sometimes unacceptably rough or even unreliable. Faced with this issue, the addition of some artificial data to a learning model in order to increase its learning accuracy is one effective approach. In virtual/synthetic data generation, the existing knowledge obtained from a given small dataset helps to create virtual samples to improve performance in regression/classification tasks.

In this research, small datasets have been utilized in both sensorial experiments. In the MSI-based case, spectral information and the related microbiological analysis from 84 beef samples were acquired, while for the e-nose-based case, a subset of used data (i.e., 58 samples) were utilized to provide volatile organic compounds (VOCs) from odor samples presented in the e-nose device. This inconsistency of data quantity in these two sensorial experiments, creates a serious problem, and needs to be addressed before applying any regression/classification techniques in the proposed fusion scheme. Thus, the first objective of this section is focused on how to create additional “virtual” microbiological data from the initial 84 beef samples, and then how to generate additional “sensorial” MSI/e-nose responses. The final goal is eventually to create a larger dataset through the usage of real/virtual data for both MSI and e-nose cases, and then to proceed to the proposed data analysis.

### 3.1. Synthetic Data Acquisition

In this work, an efficient data expansion technique was utilized for the obtained small dataset to create a new “virtual” sample set for the microbiological analysis. Inspired by the way the radial basis function (RBF) neural network approximates a nonlinear function through Gaussian local-basis functions, an RBF network was employed for each “microorganism case” (i.e., total viable counts, *Pseudomonas* spp., *Brochothrix thermosphacta* and lactic acid bacteria), utilizing the experimental microbiological data (i.e., 84 samples) as a training set [27]. Four dedicated RBF networks using the orthogonal least squares (OLS) learning algorithm have been constructed and with a smaller sampling time, through a two-inputs network, a “continuous growth curve” was obtained for each case. The RBF inputs included temperature level and sampling time-step, while the output was related to the specific microorganism predictions. Each “continuous growth curve” was verified against the real experimental data. Based on these continuous curves, 46 additional “virtual” microbiological data were obtained.

**Figure 4:**
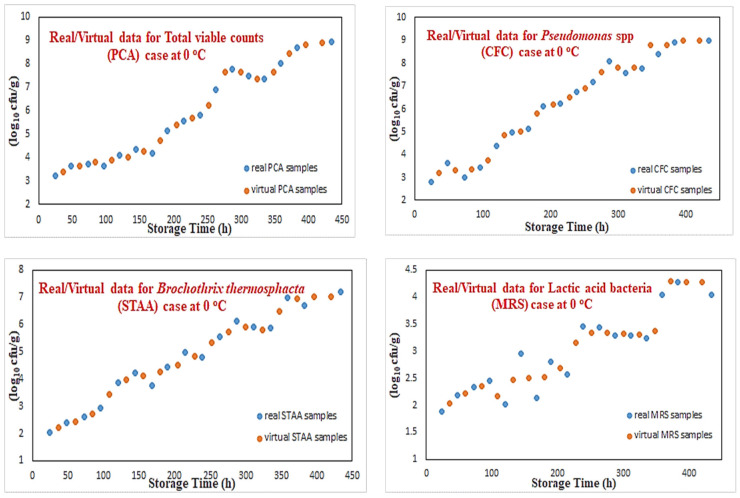
Modeling of microorganisms’ growth curves via RBF networks.

Figure 4 illustrates a sample of these growth curves for these four microorganisms at 0 °C temperature. Although in most cases there is a very close match between real and virtual data, in some other cases, some discrepancy can be noticed (such as for lactic acid bacteria). Such approximation performances can be explained by the fact that neural networks are very good models for nonlinear function approximation with good interpolating abilities. The results shown in the case of lactic acid bacteria illustrate that the generation of “virtual” data is an outcome of a learning process without the effect of data overfitting. Following this modeling procedure, a complete mixed real/virtual dataset that incorporates microbiological predictions for 130 samples has been created. This final microbiological dataset was then used to predict the class of these new “virtual” samples.

In this case, a multilayer neural network (MLP) with a two hidden layers structure was utilized. Its input vector consisted of the four “microorganisms” indicators, the temperature and time sampling, while the output node was dedicated to the class of the sample. The 84 real samples were used as the training set, while the newly acquired 46 “virtual” data as the testing set. Rather than trying to create a distinct classifier, an effort has been made to “model” the classes [28] via a regression procedure. This is an efficient and alternative way to build a classifier without the need/complexity to define, in the model, the number of classes via multiple outputs.

**Figure 5:**
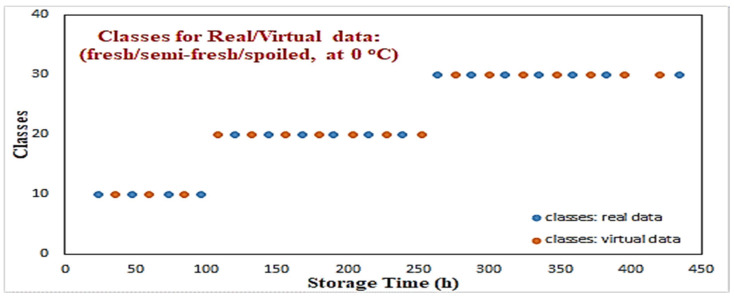
Modeling of classes for “virtual” data using an MLP neural network.

Initially, values of 10, 20, and 30 have been used, respectively, to associate the three classes with a cluster center. During the identification process, the output values in the range of [5, 15] were associated to the “fresh” class with the cluster center 10, values of [15.01, 25] were associated to the “semi-fresh” class with the cluster center 20, and finally values of [25.01, 35] were associated to the “spoiled” class with the cluster center 30. Figure 5 illustrates the classification results of these “virtual” data for the temperature of 0 °C, where a clear consistency with the real data can be observed. The creation of additional microbiological data for the “microorganisms” cases as well as the MLP-based classifier for the classes definition is considered to be a “forward modeling” process, where through some known information (i.e., time, temperature), an unknown parameter needs to be predicted (i.e., growth rates, classes).

However, the creation of “virtual” sensorial data is a more complicated task. In this paper, inspired by the inverse identification of electronic nose data [29], a similar procedure was adopted.

The MSI and e-nose outputs consist of 18 wavelengths and eight chemical sensorial responses, respectively. All these attributes are considered to be independent, in the sense that their individual responses are not dependent on the outputs of the others. Based on this assumption, a specific RBF network was employed to model each one of these attributes. In total, 26 dedicated RBF networks were trained to approximate the behavior of the devices’ outputs.

**Figure 6:**
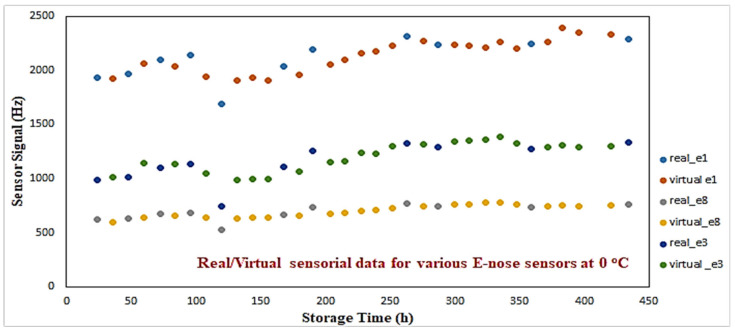
Real/virtual e-nose sensorial data for 0 °C.

The rationale of using an RBF over an MLP neural network, is that an RBF network is a scheme that represents a function of interest by using members of a family of locally supported basis functions [30]. The input vector for all RBF models included the four microbiological indicators, temperature, sampling time, and the class, whereas the e-nose/MSI outputs were considered to be the desired outputs. Based on this analysis, 72 additional “virtual” e-nose sensorial data (i.e., eight sensors per sample) and, similarly, 46 “virtual” MSI sensorial data (i.e., 18 wavelengths per sample) were created. Figure 6 illustrates the virtual/real sensorial outputs from three e-nose sensors (1st, 3rd, 8th) for 0 °C. A consistency between real vs. virtual outcomes can be observed. The aim of this procedure was to produce additional sensorial outputs that satisfactorily capture the nonlinear dynamics of the real sensorial outputs. No extreme or out-of-range responses have been monitored through this process.

**Figure 7:**
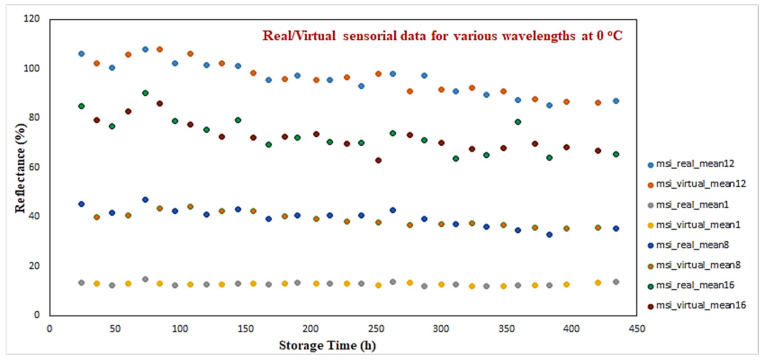
Real/virtual MSI sensorial data for 0 °C.

Similarly for the case of the MSI system, Figure 7 illustrates a sample of various wavelengths where real and virtual data are shown. More specifically, results from four (1st, 8th, 12th, 16th) wavelengths for 0 °C are illustrated. In system identification theory, the inverse modeling process is considered more challenging from the forward one, as in some cases, the inverted model cannot approximate adequately the inverse mapping of the actual process [31]. The use of RBF networks, which utilize local basis functions, provides an advantage over MLP neural networks, where a “global” approximation of the process is attempted compared to the “local” approximation of the RBF network.

**Figure 8:**
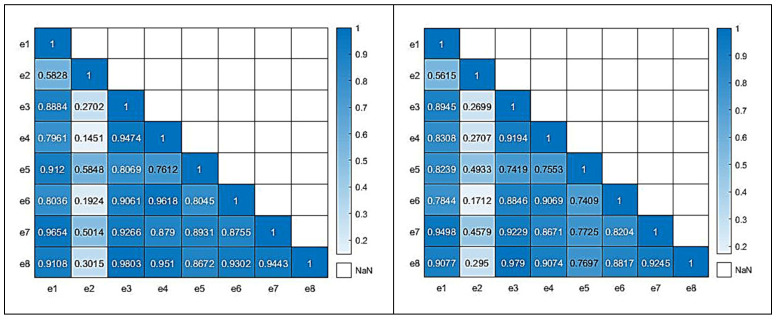
Correlation matrices (real (**left**), all data (**right**)) for the e-nose sensorial outputs.

In order to evaluate the quality of the produced virtual data, two correlation matrices of the real and all data have been produced, respectively, using MATLAB (v.2021a). Figure 8 illustrates the correlation matrices for both the real (left) and all data (right) for the case of the e-nose system. For the correlation of the real samples, 58 sensorial outputs were utilized; while, for the all-data case, the complete set of 130 samples, which included the newly acquired virtual sensorial outputs, were utilized. A close correlation score of the matrix that also includes virtual data compared to the matrix that incorporates only real data reveals a high level of consistency and validity. There are not significant deviations from the original “real” correlation matrix, which suggests that the addition of extra “virtual” data, is able to capture the complexity and behavior of the underlying data.

**Figure 9:**
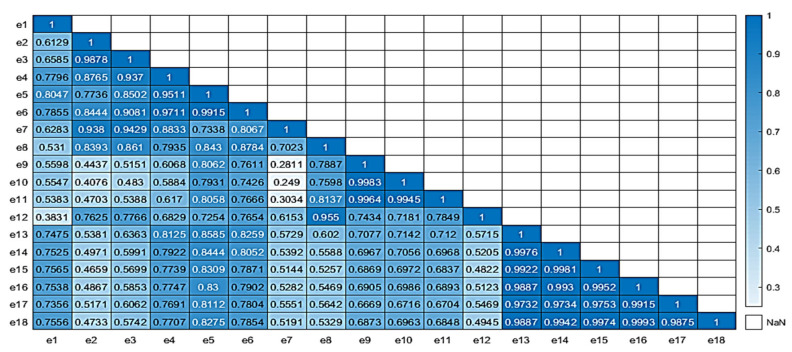
Correlation matrix of real data for the MSI wavelength outputs.

**Figure 10:**
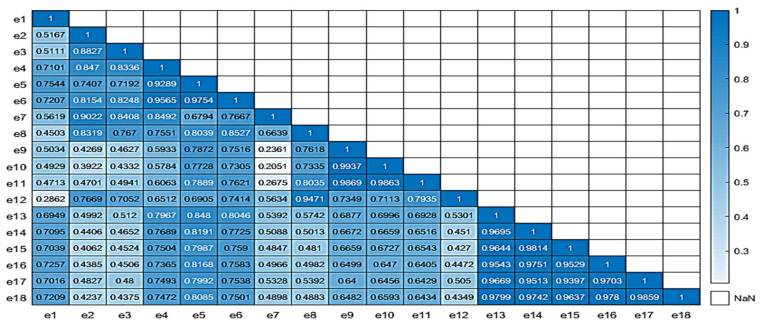
Correlation matrix of all data for the MSI wavelength outputs.

Similarly to the e-nose case, relative correlation matrices for the MSI case are shown in Figure 9 and Figure 10, revealing a similar pattern to the previous case behavior. For the correlation of the real samples, 84 sensorial outputs were utilized; while, for the all-data case, the complete set of 130 samples, which included the newly acquired virtual wavelength outputs, were utilized. The equivalent average correlation scores for real and all data were 0.723 and 0.684, respectively. Both cases reveal that the addition of the new “virtual” data did not jeopardize the quality of the overall data.

The average correlation score for the real and complete correlation matrices were 0.7603 and 0.7323, respectively, showing similar correlation characteristics.

**Figure 11:**
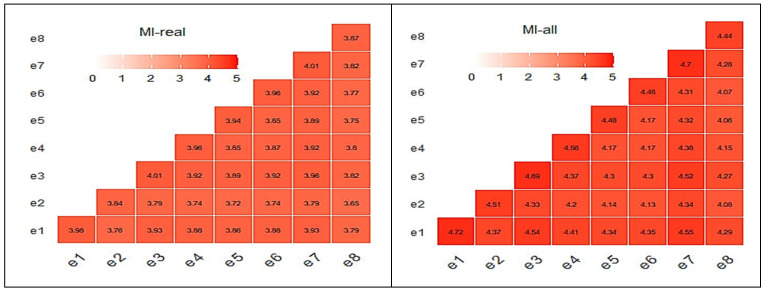
MI matrices (real (**left**), all data (**right**)) for the e-nose system.

Another useful metric to check the quality of the produced “virtual” data, is the mutual information (MI) criterion. The MI criterion measures how much knowing the value of one variable reduces the uncertainty about the value of the other. In other words, it explores how much information about one variable is contained in the other. The MI values are always non-negative, with larger values indicating a stronger relationship. In this research, the “*mutinformation*” R function from the *infotheo* R package (R version 4.2.3) has been utilized to calculate the MI for real and all data matrices. Figure 11 illustrates the MI matrices for the e-nose case. The average MI score for the real and the complete MI matrices for the case of the e-nose were 3.85 and 4.32, respectively, showing a level of resemblance. The addition of the “virtual” sensorial outputs did not have any change that could be considered to be unacceptable.

**Figure 12:**
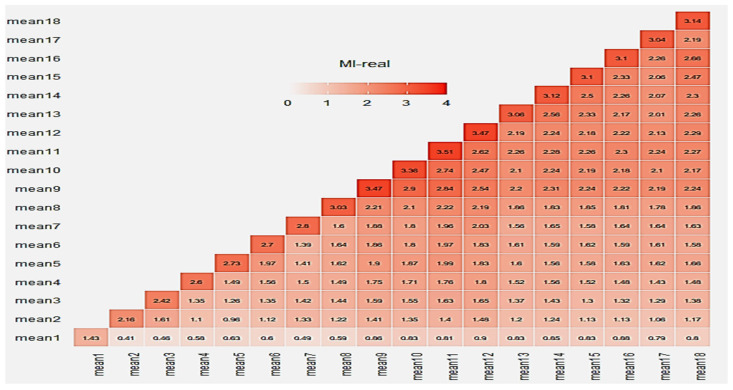
MI matrix of real data for MSI wavelength outputs.

**Figure 13:**
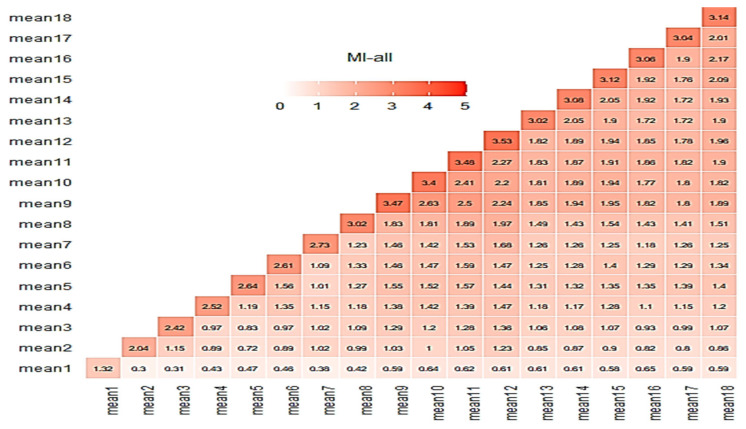
MI matrix of all data for MSI wavelength outputs.

Correlation and MI matrices seem to explain the results shown in Figure 6. Similarly to the e-nose case, relative correlation matrices for the MSI case are shown in Figure 12 and Figure 13 revealing a behavior similar to the previous case behavior, although with lower MI scores.

Following the graphs at Figure 12 and Figure 13, the related average scores for the MSI system were 1.76 and 1.46 for the real and complete data, respectively. It has to be mentioned that, for all MI cases, the “*mutinformation*” R function utilized the option to compute the entropy of the empirical probability distribution. In summary, both correlation and mutual information are ways to measure the relationship between two variables. However, they capture slightly different aspects of this specific relationship. While correlation measures the degree to which the variables move together, mutual information measures the amount of information they share.

### 3.2. Integrated Analytical Framework

**Figure 14:**
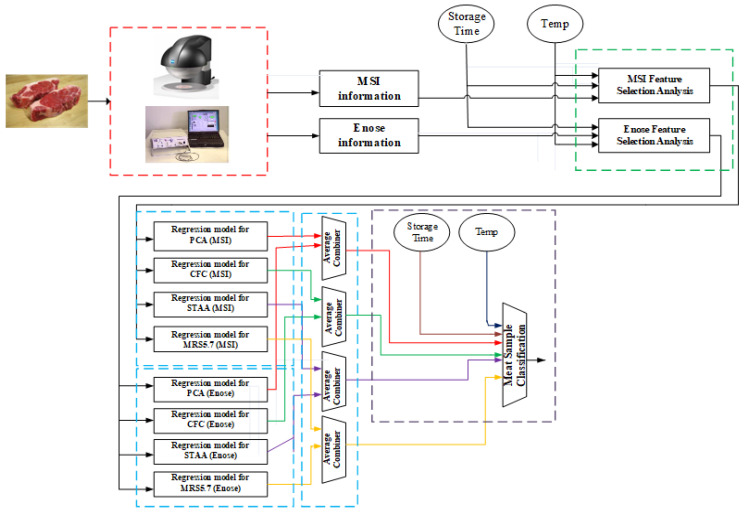
Proposed concept for data analysis.

Figure 14 illustrates the proposed analytic concept, following the imaging and volatile information acquisition from the MSI and e-nose systems, respectively. Although the schematic illustrates the various steps of analysis, in reality this framework can be realized into two stages. During the first stage, the offline part, the acquisition of spectral and volatile organic compounds together with the related microbiological analysis needs to be performed. Obviously, this is a time-consuming process, but it is required for the feature selection analysis and the training of the various regression models, and eventually for the training of the classifier which will predict the class of meat sample. The main idea of this first stage is to build offline accurate models that will diminish the need of performing microbiological analysis to each testing meat samples.

These models then could be used in the second stage, the online analysis of the meat samples, where a fast response for the quality of the meat is required. In frequent time periods, with the acquisition of new samples, the offline models can be updated/retrained and then used later in the online analysis. The acquired spectral dataset consists of the mean reflectance spectrum, while the relevant e-nose dataset includes sensorial responses expressed as frequency variations (Δf). Thus, for each meat sample, a vector of 18 wavelength attributes, eight e-nose responses, and information related to storage time and temperature was considered to be its input “signature”.

One essential issue in data analysis is to determine which actual sensorial attributes could be considered important, thus reducing the initial high-dimensional feature space provided by the sensorial systems. In the proposed framework, the Boruta feature selection (FS) method has been adopted, which is a well-known FS technique, and has been implemented around the random forest (RF) algorithm.

The development of regression models that incorporate all available information, is the next step of the analysis. The main aim at this stage is to associate the sensorial information, the storage time, and the temperature with the outcomes of the microbiological analysis. As illustrated in Figure 14, for each sensorial system and for each meat indicator, an individual regression model based on neuro-fuzzy principles has been implemented. Neuro-fuzzy models as learning systems incorporate into their “knowledge” information derived from their interpolation abilities. This is an advantage of such systems compared to traditional microbiological models which are built based on specific temperature information. Associated regression predictions are then combined through an average fusion approach in order to produce the final predictions for each meat indicator. These predictions together with information from the storage time and temperature are then utilized in a simple PLSR model to predict the class of meat samples.

## 4. Data Analysis Methodologies

### 4.1. Features Selection Scheme

The Boruta algorithm belongs to the wrapper family of features selection methods, and is implemented around the random forest (RF) technique [32]. The RF algorithm is based on the combination (or ensemble) of many decision trees. Random sampling of either dataset or input features are the main characteristic of this scheme.

**Figure 15:**
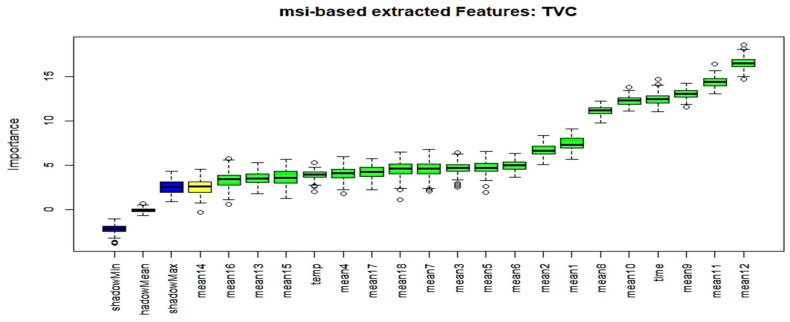
Boruta extracted features for the total viable counts (MSI case).

**Figure 16:**
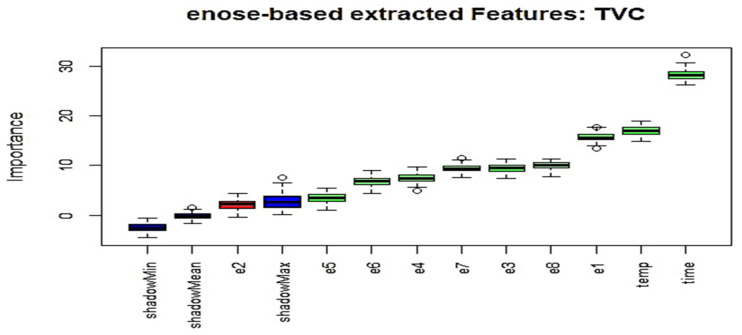
Boruta extracted features for the total viable counts (e-nose case).

The Boruta algorithm is based on the same concept, and by adding randomness to the model and gathering results from the ensemble of randomized samples, the influence of random fluctuations and correlations that could result in a false outcome can be reduced. During its operation, the so-called shadow attributes are added in the original dataset, as the focus is to check the significance of real predictor variables against these shadow attributes through statistical testing and several runs of the RF algorithm. Figure 15 illustrates the results for wavelength attributes acquired from the MSI system, as well as the storage time and temperature parameters, revealing the importance of their related attributes; while, in Figure 16, the related Boruta-based results are shown for the case of the e-nose. For both cases, the newly created complete dataset, comprising 130 samples was utilized. The columns in green are considered to be important attributes, while the ones in red are not. Some attributes, shown with a yellow color, are considered to be “uncertain”. In these two figures, the three blue bars have been introduced by the algorithm itself and represent shadowMax, shadowMin, and shadowMean attributes.

The specific analysis shown in Figure 15 is associated with the total viable counts case, utilizing spectral information from the MSI system. Although the time parameter is considered to be an important factor, temperature, on the other hand, receives much less confidence. Similarly, results from the equivalent dataset for the total viable counts for the e-nose case are illustrated in Figure 16. A significant difference in this case is that both time and temperature parameters are considered potentially to be the best features.

The Boruta algorithm was implemented in R via the related Boruta R-package (R version 4.2.3). The user can adjust the strictness of the algorithm by adjusting the *p* values, that default to 0.01, and the maxRuns, which is the number of times the algorithm is run. The higher the maxRuns the more selective you are in picking the variables. The default value is 100, and has been used in this study. Table 3 summarizes all the results related to both the e-nose and the MSI cases. Illustrated results reveal that only six e-nose features were most important, while seven wavelengths were considered to be the most useful attributes for the case of the MSI system. The selected wavelengths from the Boruta scheme “verifies” the suggestion extracted from [25], as the most important information is mainly provided from the wavelengths located at the visible part of the MSI system.

### 4.2. Adaptive Fuzzy Logic System (AFLS)

**Figure 17:**
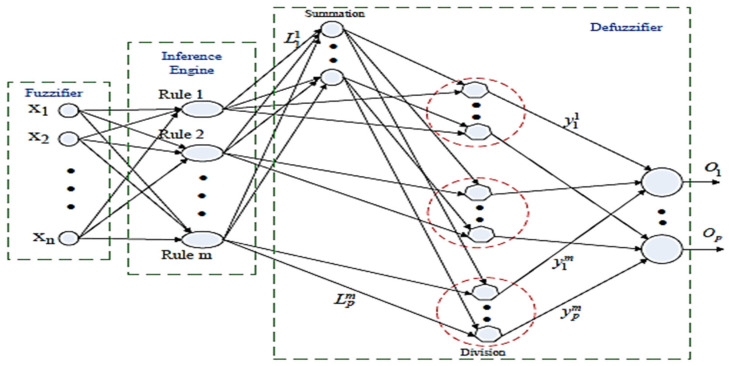
Schematic for the AFLS architecture.

Fuzzy logic systems represent the imprecision found in real-world problems using if/then rules expressed in a natural language. Although neural networks (NNs) have good learning abilities, fuzzy logic systems lack such characteristics. The main idea in neuro-fuzzy (NF) systems is to merge the capabilities of model-free and trainable systems and the noise tolerance of NNs with the ability of dealing with imprecise situations from the fuzzy set theory. ANFIS is a classic example of the NF approach, where the number of fuzzy rules is related to the number of input variables as well as the number of membership functions for each input. The proposed AFLS scheme does not follow ANFIS’s architecture, as the number of memberships for each input variable is directly associated to the number of rules; hence, the “curse of dimensionality” problem that usually appears in the ANFIS scheme is significantly reduced. In addition, AFLS utilizes a defuzzification approach, namely area of balance (AOB), which tries to resemble the well-known, but computationally expensive, centroid of area (COA) defuzzification scheme [33]. The AFLS structure is illustrated at Figure 17. AFLS’s parameters can be trained like an NN approach; however, the overall structure follows the process of a fuzzy logic system.

The first layer is the fuzzification layer and its nodes represent the fuzzy sets used in the antecedent parts of the fuzzy rules. A fuzzification node receives an input and determines the degree to which this input belongs to in the node’s fuzzy set. The outputs of this layer are the values of the “Gaussian-shape” membership functions (MF) for the input values.

(1) $$\mu_{F_{i}^{m}}(x_{i})=\exp\left[-\frac{{\left(x_{i}-c_{i}^{m}\right)}^{2}}{2{\left(b_{i}^{m}\right)}^{2}}\right]$$

where $c_{i}^{m}$ and $b_{i}^{m}$ are the center and spread parameters of the membership function for the ith input and the mth rule. The next layer is the inference layer, which is related to the rules’ firing strength. Since each fuzzy rule’s antecedent part has an AND connection operator, in this paper, the firing strengths are calculated using the product T-norm operator (i.e., multiplication). Thus, the output of this layer has the following form:

(2) $$\mu_{m}(\bar{x})=\prod_{i=1}^{n}\mu_{F_{i}^{m}}(x_{i}),\;m=1,2,\ldots ,M$$

where $\mu_{F_{i}^{m}}(x_{i})$ is the membership value of the *i*th input of rule m. The number of rules at this layer is equal to the number of MFs allocated to each input variable, thus minimizing the problem of the excessive number of rules usually encountered in the case of the ANFIS model. The most popular defuzzification methods in fuzzy logic systems are the centroid of area (COA) and center average (CA) schemes. Although more accurate than the latter, the former is well known for its computational cost. Centroid calculation returns the centroid of the area formed by the consequent membership function, the membership value of its rules and the max–min or max- product inference. Some of the COA’s main characteristics, such as the center of gravity and the use of the shape of membership function, have been adopted in the proposed AOB scheme [33].

In general, the calculation of the output, *y*, is defined as follows:

(3) $$y_{p}=\frac{\sum_{m=1}^{M}\mu_{m}L_{p}^{m}y_{p}^{m}}{\sum_{m=1}^{M}\mu_{m}L_{p}^{m}}$$

where *y_p_* is the *p*th output of the network, *μ_m_* is the membership value of the *m*th rule, $L_{p}^{m}$ is the spread parameter of the membership function in the consequent part of the *p*th output of the *m*th rule, and $y_{p}^{m}$ is the center of the membership function in the consequent part of the *p*th output of the *m*th rule. The gradient descent learning algorithm was applied for the training phase in order to update its various parameters. The update equations for $y_{p}^{m}$, $L_{p}^{m}$, $c_{i}^{m}$, and $b_{i}^{m}$ are as follows:

(4) $$y_{p}^{m}(n+1)=y_{p}^{m}(n)+m_{y}[y_{p}^{m}(n)-y_{p}^{m}(n-1)]-\eta_{y}\frac{\partial J}{\partial y_{p}^{m}}{|}_{n}$$

(5) $$L_{p}^{m}(n+1)=L_{p}^{m}(n)+m_{L}[L_{p}^{m}(n)-L_{p}^{m}(n-1)]-\eta_{L}\frac{\partial J}{\partial L_{p}^{m}}{|}_{n}$$

(6) $$c_{i}^{m}(n+1)=c_{i}^{m}(n)+m_{c}[c_{i}^{m}(n)-c_{i}^{m}(n-1)]-\eta_{c}\frac{\partial J}{\partial c_{i}^{m}}{|}_{n}$$

(7) $$b_{i}^{m}(n+1)=b_{i}^{m}(n)+m_{b}[b_{i}^{m}(n)-b_{i}^{m}(n-1)]-\eta_{b}\frac{\partial J}{\partial b_{i}^{m}}{|}_{n}$$

where, *J_k_* the objective function is defined as follows:

(8) $$J_{k}=\frac{1}{2}\sum_{p=1}^{P}(y_{p}({\bar{x}}_{k})-d_{p}({\bar{x}}_{k}){)}^{2}$$

with P is the number of outputs, *d_p_* is the desired response of the *p*th output, and $y_{p}({\bar{x}}_{k})$ is defined as in Equation (3). The AFLS architecture has been implemented in MATLAB (ver. R2021a, Mathworks.com).

## 5. Learning Models for Regression and Classification Tasks

The main objective in this paper is the efficient estimation of the level for each of these specific” microorganism cases” (i.e., total viable counts, *Pseudomonas* spp., *Brochothrix thermosphacta* and lactic acid bacteria) through the fusion of the MSI and e-nose systems. In addition to selected sensorial outputs, information related to specific time-steps at which beef samples were analyzed during storage and temperature levels was considered to be an additional input to the various employed learning models. A follow-up objective is to predict the class of testing meat samples to their “quality” class (i.e., fresh, semi-fresh, spoiled). A simple PLSR model has been employed to predict the “class” of these samples, receiving as inputs, information from temperature, time storage, and the predicted fusion estimations for each microorganism case. One of the key elements of the proposed framework, shown in Figure 14, are the regression models to estimate related “microorganism” cases. The AFLS scheme has been employed as a regression model, while the obtained results are compared with those obtained by the MLP neural networks, support vector machines (SVM), extreme gradient boosting (XGBoost), and PLSR models. The MLP, SVM, and XGBoost algorithms are considered very popular in the area of machine learning, while PLSR has been extensively used in food microbiological applications. In fact, PLSR’s low complexity, and its ability to provide generally satisfactory results, have attracted the interest of researchers in this specific application field.

The final dataset for each sensorial case (MSI/e-nose) consisted of 130 meat samples, which also incorporated the additional “virtual” generated samples. For each sample (real/virtual), sensorial outputs, storage time and temperature information, type of class, and the estimation of the level for each of these specific “microorganism cases” has been available. In this research study, two schemes have been considered for the training/testing stages. In the first scheme, the initial (MSI/e-nose) datasets were divided into training subsets with approx. 89% of the data and testing subsets with the remaining 11% (i.e., 15 samples). For these testing subsets, only real samples/sensorial responses were considered. These 15 testing samples were common in both the MSI and e-nose experiment and, for each temperature, three representative samples were selected. More specifically, samples at 48, 168, and 359 h time steps were chosen for 0 °C, at 24, 12, and 311 h for 4 °C, at 24, 69, and 175 h for 8 °C, at 16, 48, and 100 h for 12 °C and at 12, 36, and 77 h for 16 °C. In total, five testing samples were allocated to each meat class. For the second training/testing scheme, the leave-one-out cross-validation (LOOCV) has been employed. This is a well-known procedure applied in cases where the number of observations/samples is small, and the separation of the dataset into training and testing subsets is considered that it would result in insufficient training of the leaning model [34].

The performance of these learning regression models has been evaluated through a number of well-established metrics. The measure of goodness-of-fit for model comparison in food microbiology is performed with the squared correlation coefficient (R^2^). This metric can be explained as the ratio of the variance of the predicted responses from the objective related responses. It is considered to be a suitable evaluation criterion only under the condition that the error is normally distributed and not dependent on the mean value. In reality, for the case of bacteria growth, the distribution of the error is not clearly known, thus this metric should be used with caution, particularly in nonlinear-based regression models [35]. Similarly to general purposes regression case studies, evaluation metrics, such as the mean absolute percentage error (MAPE), the root mean squared error (RMSE), the standard error of prediction (SEP), the absolute percentage error (APE), and the mean absolute error (MAE) metrics, have also been explored in this research. Finally, three chemometric metrics, which are used extensively in spectroscopic applications, namely residual prediction deviation (RPD), range error ratio (RER), and the ratio of performance to interquartile distance (RPIQ), were also utilized in this paper. The RPD is calculated as the ratio of the standard deviation of the desired variable to the RMSE. Any model with an RPD value above three is generally assumed to be an excellent model in terms of reliability. The ratio of performance to interquartile distance (RPIQ), which is defined as an interquartile range of the observed values divided by the RMSE, is considered to be a metric of model validity that is more objective than the RMSE. A larger RPIQ value is considered to be desirable. Finally, the range error ratio (RER) is equal to the range in the observed values (i.e., the maximum value minus the minimum value) divided by the RMSE. Any RER value above 10 is considered to be desirable [36].

### 5.1. “Regression” Task

All regression models for each microorganism case have been implemented utilizing the same input vector which is illustrated in Table 3. Initially, models were developed based on the reduced (115/15) dataset scenario. Using a trial and error method, it has been found that the chosen number of fuzzy rules was ranged between 10 and 16 for these AFLS models used to approximate the specific microorganism case for each sensorial system. The number of membership functions for each input variable is directly associated with the number of rules; hence, each input signal is “distributed” through Gaussian functions with different centers and widths to every rule node via a product operator. The values of the parameters (centers and widths) of the Gaussian membership functions have been adjusted by the gradient descent (GD) learning algorithm that was utilized as a learning scheme. Although the AFLS scheme shared with the MLP model the same learning algorithm, the training time was completed in less than 1000 epochs, much faster from the equivalent time used to train the MLP neural network.

**Figure 18:**
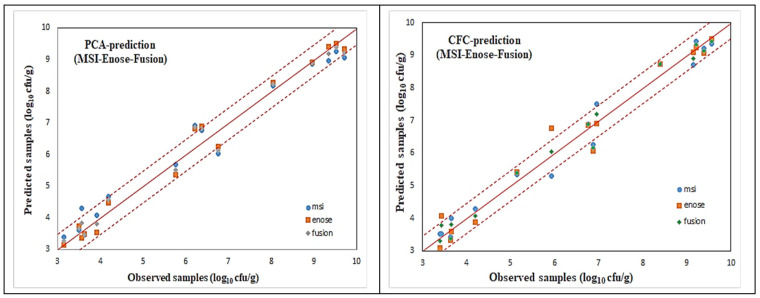
AFLS prediction models for PCA and CFC cases.

**Figure 19:**
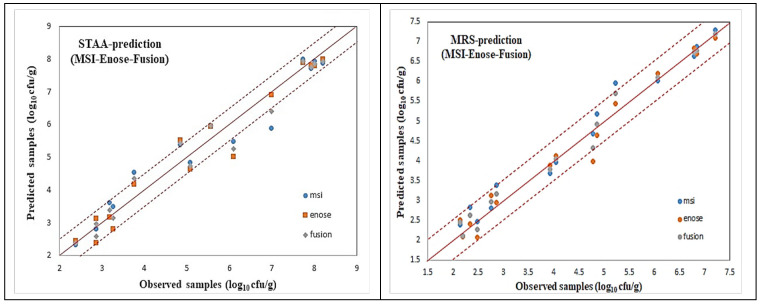
AFLS prediction models for STAA and MRS cases.

For all these microorganism cases, illustrated via the name of the agar medium used, scatter plots of the predicted (via AFLS) vs. the observed testing samples for the reduced case are illustrated in Figure 18 and Figure 19, which reveal a very good distribution around the line of equity (y = x), with the vast majority of the data included within the ±0.5 log area, especially for the fusion (i.e., average) scheme.

Even though the fusion scheme seems to be more accurate, it is interesting to notice the performances of the e-nose and MSI cases from these figures. It is rather difficult to confirm which sensorial device is the winner. This is due to the fact that the e-nose sensors and MSI wavelengths “capture” different characteristics of the same meat sample. Such diversity in the acquired results from the MSI and the e-nose is evidence of the validity of the sensor fusion hypothesis.

The testing performance of all the developed models to predict the above mentioned microorganism cases in the beef samples, in terms of statistical indices, are presented in Table 4 and Table 5. Even though fusion (via averaging) results for all the microbiological cases are superior, it is interesting to notice that the obtained individual per device results are acceptable, by checking especially the chemometric metrics for RPD, RER, and RPIQ. Such values reveal a clear robustness of the produced AFLS models.

Traditionally, in machine learning based applications, a common practice is to compare any proposed algorithm against other well-established methods in order to validate the chosen approach. In this research, four alternative regression models have also been developed. An MLP network utilizing two hidden layers, as well as a partial least squares regression (PLSR) scheme have also been implemented, due to the fact that these two schemes have been widely utilized in such applications. Additionally, support vector machine (SVM) and extreme gradient boosting (XGBoost) models, widely used in machine leaning, have been added due to the fact these specific algorithms are usually considered to be “competitors” to neural networks. The performance of all these models to predict the above mentioned microorganism cases for both the MSI and e-nose scenarios, in terms of statistical indices, are presented in Table 6, Table 7, Table 8 and Table 9.

The performance of the classic MLP neural network can be considered to be acceptable, although inferior to the related AFLS performance. Especially for the e-nose/STAA case, the MLP model, built with the backpropagation learning algorithm, achieved a rather comparable performance against the equivalent AFLS model. However, such performances were achieved with a relative high computational cost (more than 10,000 epochs). A two hidden layers structure was adopted for all models, while the number of nodes in the first and second hidden layers were ranged between 16 and 20 and 8 and 12, respectively. Overall, both the AFLS and MLP models revealed their robustness in predicting such complex dynamic characteristics as these microorganisms, regardless of the sensorial device used, proving that these learning architectures can be considered to be general purpose learning schemes.

A support vector machine (SVM) is a powerful machine learning approach based on statistical learning theory. Its advantages over the MLP models include a global optimal solution and robustness to outliers. In the context of regression, SVM aims to find a hyperplane that maximizes the margin between the predicted values and the actual values. It utilizes the so-called support vectors, which are the data points closest to the hyperplane, to define the regression line. The specific SVM used in this research involves epsilon support vector regression (ε−SVR). The value of epsilon is used to measure the error between the predicted and the real values in a high-dimension space, and its value is determined based on practical experience. For this specific case study, SVR models were implemented in R using the e1071 R package. The penalty coefficient C was ranged to values >200, the gamma (γ) parameter that controls the smoothness of the decision boundary in the feature space was set to values >0.01, while the epsilon tolerance value was set to values >0.03. The results shown in Table 6, Table 7, Table 8 and Table 9 reveal an inferior regression performance over the MLP network; however, these results could be considered to be acceptable as the calculated RPD was greater than three. Similarly to the MLP case, the SVM revealed a rather stable performance regardless of the sensorial device used.

The XGBoost algorithms belong to the group of ensemble learning, specifically boosting, where multiple weak learners (typically decision trees) are jointly used to create a robust and accurate predictive model [37]. The goal is to minimize a loss function, and each weak learner is projected to correct the errors in the present ensemble. The algorithm’s strength stems from its ability to balance predictive accuracy and regularization, making it flexible and suitable for a wide range of machine learning applications. In this research, the XGBoost R package was employed to apply this algorithm for this application. The results shown at Table 6, Table 7, Table 8 and Table 9 reveal some diversity in terms of the sensorial device used and the specific microorganism case. Generally speaking, the application of XGBoost to the MSI cases was disappointed. A very low RPD value, along with the other metrics, proved the difficulty of this algorithm to handle this specific regression application. On the other hand, the e-nose application was much more improved, especially with the cases of PCA and STAA, where the algorithm really achieved a remarkable performance. Finally, a partial least squares regression (PLSR) scheme was applied to the same datasets. The PLS models were constructed using the same input vectors as the previous models, while the PLS_Toolbox (v. R9.1) software in association with MATLAB was used to perform the PLS analysis. The SIMPLS algorithm was chosen as the appropriate optimization scheme. The algorithm calculates the PLS factors directly as linear combinations of the original variables. These factors are determined so as to maximize a covariance criterion, while obeying certain orthogonality and normalization restrictions. It is well known that, in the modeling of real complex processes, linear PLSR has some difficulties, since most real problems are inherently nonlinear and dynamic. Although the method has a very low computational cost, and has wide applicability in food microbiological applications, the obtained results in all cases were rather disappointing. A close inspection at these results reveals that, for the case of the PLSR scheme, the obtained RPD values revealed that such a model cannot be considered to be acceptable/preferable.

Additionally, for each microorganism case, the proposed AFLS architecture was implemented in a LOOCV scenario for both the MSI and the e-nose components. Table 10 and Table 11 illustrate the related results via the same performance indices. Similar to the previous scenario, even in the LOOCV scheme, the proposed average fusion scheme outperformed the individual sensorial performances.

In summary, these regression results justified the need for advanced learning methods to regression tasks related to food analysis. The generic algorithms in machine learning, like MLP and SVM, proved their suitability, even if they have been outperformed by the hybrid neuro-fuzzy models. However, it was a surprise to see algorithms well known for their regression capabilities, like XGBoost, produce such diverse experience. One of the lessons learned by this research is that we always need to provide “tailored” solutions to specific problems. Unfortunately, in such types of applications, it is also rather restricted to accommodate an enormous amount of experimental data. Therefore, researchers need to search for methodologies that will enable them to create additional “virtual” data. In this research, through the use of RBF neural networks, additional data (microbiological as well as sensorial) were created, keeping the number of available temperatures constant. It might be interesting, in a future work, to investigate the creation of additional data in an expanded range of temperatures, and then to explore how a set of experimental data in a given temperature will perform in a regression task (through the interpolation abilities of learning based systems).

### 5.2. “Classification” Case Study

The final step in the proposed analytic framework is related to the identification of the class of testing meat samples. For this specific step, a simple PLSR scheme was employed in order to predict the type of class (i.e., fresh/semi-fresh/spoiled) of meat. This PLSR scheme was applied in the reduced scenario case (115 training vs. 15 testing samples respectively). The input vector consisted of the final four microorganism prediction levels (PCA, CFC, STAA and MRS) after the fusion, storage time, and temperature while the output of the regression model corresponded to the three-class cases (10, 20, and 30) which correspond to fresh/semi-fresh/spoiled classes. Details of the proposed PLSR scheme is shown in the following equation:

(9) $$\begin{array}{ll} class= & -0.954-0.0195*time-0.1624*temp+1.591*PCA\\ & +2.432*CFC+1.903*STAA-2.2963*MRS \end{array}$$

Table 12 shows the related PLSR results on the testing dataset. It is clear that a 100% classification rate has been achieved, thereby verifying the validity of the framework concept shown at Figure 14.

Although, in this specific case study, only two individual sensing devices were utilized and their subsequent fusion was based on a simple average scheme, such a concept could be easily extended to include additional sensors and thus more advanced fusion strategies could be applied.

## 6. Conclusions

In this research, a proposed machine learning based framework for the detection of meat spoilage through the fusion of MSI and e-nose information has been investigated. The limitation of small size available datasets was addressed with the generation of additional “virtual” microbiological and sensorial data through the use of radial basis function neural networks. Feature selection analysis for each sensorial device was performed via the Boruta algorithm, while the AFLS NF regression models were employed to approximate each microorganism case through a high level fusion scheme. The performance of the AFLS models was evaluated through a number of established metrics, and compared successfully against the MLP, SVM, XGBoost, and PLSR models. Finally, a simple PLSR model was employed to predict the type of testing for the meat samples into three distinct classes, namely fresh, semi-fresh, and spoiled. Even though the performance of the implemented analytical framework was great, a number of open issues still remain. Future work will concentrate on modifying the existing analytical framework by incorporating additional sensorial information, such as FTIR, and utilizing an ensemble stacking model instead of the classic average fusion scheme. Although it achieved a robust performance, the AFLS model needs to be modified, as currently the number of fuzzy rules are chosen by the user. It would be interesting to automate this structural process by introducing a clustering component that will determine the number of input memberships/fuzzy rules. Neural networks are considered to be the most advanced techniques of automated data generation. They can handle much richer data distributions than traditional algorithms, such as decision trees. Although currently, an RBF neural network was utilized as the data generator, alternative algorithms based on neural network principles, such as variational autoencoders and generative adversarial networks, could provide an alternative solution. Obviously, the application domain can be expanded by investigating a freshness assessment for pork, poultry, and fish products. Similarly, the quality evaluation of fruits is of particular interest, through the use of multiple sensorial devices. Finally, authentication and control of adulteration are crucial for the meat and olive oil industry to ensure levels of quality for their products, as well as to safeguard the health and safety of consumers.

## Figures and Tables

**Table 1:** Categorization of growth ranges for all meat indicators.

Class	Temp	PCA	CFC	STAA	MRS
	°C	(log_10_CFU/g)	(log_10_CFU/g)	(log_10_CFU/g)	(log_10_CFU/g)
Fresh (F)	0	3.208–3.742	2.809–3.643	2.048–2.920	1.887–2.444
	4	3.146–3.712	3.397–4.375	2.866–3.091	2.479–2.426
	8	3.058–4.336	2.815–4.665	2.236–3.796	1.989–2.785
	12	3.502–4.514	3.042–4.994	1.864–4.235	2.516–3.727
	16	3.566–3.807	3.544–3.652	2.791–2.859	2.617–2.861
Semi-fresh (SF)	0	4.099–5.818	4.40–6.748	3.761 4.975	2.012–3.455
	4	3.507–7.005	4.77–7.204	3.544–6.892	2.61–4.707
	8	5.688–6.762	5.364–7.151	4.324–6.096	3.672–4.879
	12	4.989–7.854	5.284–8.047	4.364–6.531	4.042–5.579
	16	6.263–6.371	6.229–6.948	5.397–5.566	4.919–5.232
Spoiled (S)	0	6.907–8.947	7.163–9.002	5.55–7.187	3.237–4.284
	4	7.76–9.884	8.063–9.753	6.393–8.038	4.416–6.261
	8	7.241–9.539	7.116–9.512	6.161–8.206	4.896–7.04
	12	7.947–9.345	7.907–9.335	7.031–8.117	5.898–7.035
	16	7.453–9.714	7.402–9.459	6.704–8.037	5.784–7.924

**Table 2:** Active matrix used to coat the QCM sensors of the Libra e-nose.

Sensor	Aldehydes	Polymers–Derivatives
1	Phenanthrene-9-aldehyde	Poly[2-(9-phenanthryl-ylmethyl)]-1*H*-pyrrole
2	*trans*-cinnamaldehyde	Poly{2-[2-(2E)-3-phenylprop-2-enyl]}-1*H*-pyrrole
3	Ferrocene carboxaldehyde	Poly(ferrocene)-1*H*-pyrrole
4	4-ethoxy-3-hydroxybenzaldehyde	Poly[(2-ylmethyl)-2-2ethoxyphenol]-1*H*-pyrrole
5	Benzaldehyde	Poly[2-(benzyl)]-1*H*-pyrrole
6	Thiophene-2-carboxyaldehyde	Poly[2-(thien-2-ylmethyl)]-1*H*-pyrrole
7	1-acethyl-3-indole carboxaldehyde	Poly[1-acetyl-1*H*-indole]-1*H*-pyrrole
8	Anisaldehyde	Poly[2-4(methoxybenzyl)]-1*H*-pyrrole

**Table 3:** Summary of the selected by Boruta Attributes.

Sensors (Meat Indicators)	Selected Features (*Sorted by Importance Levels*)
E-nose: Total Viable Counts (PCA)	time, temp, e1, e8, e3, e7, e4, e6
E-nose: *Pseudomonas* spp. (CFC)	time, temp, e1, e8, e7, e3, e4, e6
E-nose: *Brochothrix thermosphacta* (STAA)	time, temp, e1, e8, e3, e7, e4, e6
E-nose: Lactic Acid Bacteria (MRS)	temp, time, e1, e8, e3, e7, e4, e6
MSI: Total Viable Counts (PCA)	m12, m11, m9, time, m10, m8, m1, m2
MSI: *Pseudomonas* spp. (CFC)	m12, m11, time, m9, m10, m8, m1, m2
MSI: *Brochothrix thermosphacta* (STAA)	m12, m11, m9, m10, time, m8, m2, m1
MSI: Lactic Acid Bacteria (MRS)	m12, temp, m11, m8, m9, m10, time, m4

**Table 4:** Performance of developed AFLS models for the total viable counts and *Pseudomonas* spp.

	PCA (Agar Medium)			CFC (Agar Medium)		
Statistical Indices	MSI/AFLS	E-Nose/AFLS	Fusion	MSI/AFLS	E-Nose/AFLS	Fusion
*Root mean squared error (RMSE)*	0.4308	0.3274	0.3403	0.3435	0.4020	0.2816
*Mean absolute percentage error (MAPE)*	6.5593	4.9241	5.0147	4.8042	5.9302	4.1374
*Standard error of prediction (SEP %)*	6.9755	5.3001	5.5088	5.3841	6.3013	4.4150
*APE*	98.3894	73.8616	75.220	72.0636	88.9525	62.0615
*MAE*	0.3577	0.2683	0.2841	0.2912	0.3037	0.2348
*Residual Prediction Deviation (RPD)*	5.6951	7.4953	7.2113	6.8991	5.8948	8.4133
*Range Error Ratio (RER)*	15.2440	20.0626	19.3024	17.9403	15.3289	21.8781
*Ratio of Performance* to *Interquartile distance*	11.6884	15.3831	14.8002	15.0470	12.8568	18.3497
*R-squared (R* ^2^ *) index*	0.9866	0.9905	0.9903	0.9887	0.9847	0.9924

**Table 5:** Performance of developed AFLS models for *Brochothrix thermosphacta* and lactic acid bacteria.

	STAA (Agar Medium)			MRS (Agar Medium)		
Statistical Indices	MSI/AFLS	E-Nose/AFLS	Fusion	MSI/AFLS	E-Nose/AFLS	Fusion
*Root mean squared error (RMSE)*	0.4562	0.4304	0.3949	0.2992	0.2906	0.2343
*Mean absolute percentage error (MAPE)*	7.1527	7.4047	6.4982	6.2377	6.1811	5.5705
*Standard error of prediction (SEP %)*	8.6887	8.1971	7.5206	6.9581	6.7587	5.4489
*APE*	107.2905	111.071	97.4723	93.5653	92.7165	83.5570
*MAE*	0.3575	0.3373	0.3240	0.2159	0.2128	0.1803
*Residual Prediction Deviation (RPD)*	4.6746	4.9549	5.4007	6.0966	6.2764	7.7852
*Range Error Ratio (RER)*	12.7553	13.5202	14.7365	16.9449	17.4447	21.6383
*Ratio of Performance* to *Interquartile distance* (*RPIQ*)	9.5077	10.0779	10.9845	11.0430	11.3687	14.1016
*R-squared (R* ^2^ *) index*	0.9761	0.9789	0.9820	0.9877	0.9865	0.9915

**Table 6:** Performance of machine learning models for the total viable counts.

Statistical Indices (PCA)	MSI/MLP	E-Nose/MLP	MSI/SVM	E-nose/SVM	MSI/XGB	E-Nose/XGB	MSI/PLS	E-Nose/PLS
*RMSE*	0.4457	0.4872	0.5764	0.5943	1.0214	0.4699	1.2866	1.3004
*MAPE*	7.0985	7.8585	10.1085	8.8193	15.5995	6.8770	21.9661	20.9216
*SEP*	7.2155	7.8871	9.3320	9.6212	16.537	7.6078	20.8308	21.0542
*APE*	106.4782	117.8780	151.6279	132.2893	233.9923	103.1557	329.4915	313.8234
*MAE*	0.3852	0.3839	0.4959	0.4506	0.7404	0.3704	1.1137	1.0281
*RPD*	5.5057	5.0369	4.2570	4.1290	2.4022	5.2218	1.9071	1.8869
*RER*	14.7370	13.4820	11.3946	11.0521	6.4301	13.9770	5.1046	5.0504
*RPIQ*	11.2996	10.3374	8.7368	8.4742	4.9303	10.7169	3.9140	3.8725
*R-squared (R* ^2^ *)*	0.9858	0.9802	0.9766	0.9691	0.9097	0.9805	0.8754	0.7018

**Table 7:** Performance of machine learning models for *Pseudomonas* spp.

Statistical Indices (CFC)	MSI/MLP	E-Nose/MLP	MSI/SVM	E-Nose/SVM	MSI/XGB	E-nose/XGB	MSI/PLS	E-Nose/PLS
*RMSE*	0.4077	0.4748	0.5612	0.6457	0.8082	0.5397	1.2042	1.3235
*MAPE*	6.9344	7.6559	9.1870	10.7023	12.1288	7.3531	20.1532	19.0529
*SEP*	6.3909	7.4438	8.7976	10.1217	12.6693	8.4612	18.8773	20.7480
*APE*	104.0153	114.8391	137.8048	160.5343	181.9317	110.2960	302.2983	285.7942
*MAE*	0.3360	0.3698	0.4941	0.5069	0.6007	0.4478	1.0098	0.9391
*RPD*	5.8122	4.9901	4.2222	3.6699	2.9319	4.3901	1.9677	1.7903
*RER*	15.1140	12.9763	10.9794	9.5431	7.6242	11.4160	5.1169	4.6555
*RPIQ*	12.6765	10.8836	9.2087	8.0041	6.3946	9.5749	4.2916	3.9047
*R-squared (R* ^2^ *)*	0.9889	0.9791	0.9741	0.9602	0.9474	0.9745	0.8867	0.8197

**Table 8:** Performance of machine learning models for *Brochothrix thermosphacta*.

Statistical Indices (STAA)	MSI/MLP	E-Nose/MLP	MSI/SVM	E-Nose/SVM	MSI/XGB	E-Nose/XGB	MSI/PLS	E-Nose/PLS
*RMSE*	0.5118	0.4609	0.6323	0.5182	0.9828	0.6154	1.1933	1.1775
*MAPE*	8.8372	8.5708	11.5118	8.1113	16.9262	11.4935	23.0652	20.7943
*SEP*	9.7480	8.7772	12.0431	9.8691	18.7183	11.7213	22.7277	22.4261
*APE*	132.5582	128.5623	172.6772	121.6690	253.8926	172.4024	345.9773	311.9145
*MAE*	0.4192	0.3718	0.5537	0.3910	0.7318	0.5059	0.9883	0.9073
*RPD*	4.1666	4.6275	3.3726	4.1155	2.1699	3.4652	1.7871	1.8111
*RER*	11.3692	12.6267	9.2026	11.2297	5.9208	9.4552	4.8763	4.9419
*RPIQ*	8.4745	9.4119	6.8595	8.3706	4.4133	7.0479	3.6348	3.6837
*R-squared (R* ^2^ *)*	0.9704	0.9748	0.9553	0.9726	0.8915	0.9570	0.8402	0.8217

**Table 9:** Performance of machine learning models for lactic acid bacteria.

Statistical Indices (MRS)	MSI/MLP	E-Nose/MLP	MSI/SVM	E-Nose/SVM	MSI/XGB	E-Nose/XGB	MSI/PLS	E-Nose/PLS
*RMSE*	0.3537	0.3487	0.4548	0.4077	0.7101	0.3248	0.8838	1.1389
*MAPE*	7.1321	8.8841	10.7273	9.9954	15.5532	6.5759	20.8279	26.8953
*SEP*	8.2249	8.1092	10.5769	9.4815	16.5126	7.5522	20.5536	26.4858
*APE*	106.9813	133.2616	160.9088	149.9311	233.2984	98.6381	312.4180	403.4299
*MAE*	0.2565	0.2872	0.3916	0.3374	0.5270	0.2633	0.7071	0.8834
*RPD*	5.1576	5.2312	4.0107	4.4741	2.5690	5.6170	2.0639	1.6016
*RER*	14.3351	14.5396	11.1473	12.4352	7.1402	15.6119	5.7364	4.4516
*RPIQ*	9.3421	9.4754	7.2647	8.1040	4.6533	10.1742	3.7384	2.9011
*R-squared (R* ^2^ *)*	0.9807	0.9813	0.9671	0.9764	0.9159	0.9841	0.8775	0.7734

**Table 10:** Performance of AFLS models for the total viable counts and *Pseudomonas* spp. (LOOCV).

LOOCV	PCA			CFC		
Statistical Indices	MSI/AFLS	E-Nose/AFLS	Fusion	MSI/AFLS	E-Nose/AFLS	Fusion
*Root mean squared error (RMSE)*	0.2874	0.2527	0.2641	0.2300	0.2801	0.2285
*Mean absolute percentage error (MAPE)*	3.7146	3.3232	3.4696	2.7251	3.6827	2.8026
*Standard error of prediction (SEP %)*	4.1151	3.6183	3.7819	3.2029	3.9012	3.1830
*APE*	482.901	432.012	451.051	354.2651	478.7495	364.341
*MAE*	0.2169	0.1978	0.2041	0.1754	0.2176	0.1755
*Residual Prediction Deviation (RPD)*	7.5110	8.5421	8.1726	9.0345	7.4173	9.0909
*Range Error Ratio (RER)*	23.7522	27.0132	25.8446	30.1961	24.7906	30.3843
*Ratio* of *Performance* to *Interquartile distance* (*RPIQ*)	14.5431	16.5397	15.8242	15.9582	13.1015	16.0576
*R-squared (R* ^2^ *) index*	0.9911	0.9931	0.9925	0.9939	0.9912	0.9939

**Table 11:** Performance of AFLS models for *Brochothrix thermosphacta* and lactic acid bacteria (LOOCV).

LOOCV	STAA			MRS		
Statistical Indices	MSI/AFLS	E-Nose/AFLS	Fusion	MSI/AFLS	E-Nose/AFLS	Fusion
*Root mean squared error (RMSE)*	0.2647	0.2884	0.2512	0.2038	0.1995	0.1826
*Mean absolute percentage error (MAPE)*	3.8102	4.4230	3.6768	4.0068	3.8460	3.5722
*Standard error of prediction (SEP %)*	4.4848	4.8860	4.2556	4.3433	4.2504	3.8910
*APE*	495.3237	574.9856	477.985	520.8866	499.9811	464.381
*MAE*	0.2019	0.2255	0.1938	0.1601	0.1493	0.1399
*Residual Prediction Deviation (RPD)*	6.8744	6.3100	7.2447	8.3649	8.5479	9.3373
*Range Error Ratio (RER)*	23.9570	21.9901	25.2473	29.6170	30.2648	33.0598
*Ratio* of *Performance* to *Interquartile distance* (*RPIQ*)	11.6597	10.7024	12.2877	15.2465	15.5800	17.0189
*R-squared (R* ^2^ *) index*	0.9909	0.9876	0.9905	0.9937	0.9931	0.9944

**Table 12:** Classification results for the testing dataset.

Time	Temp	PCA	CFC	STAA	MRS	Desired Class	PLSR Prediction	PLSR Result (Final)
48	0	3.47515	3.3747	2.3816	2.1003	**10**	11.559	10
168	0	4.5838	5.38975	4.34245	2.45425	**20**	18.805	20
359	0	8.21335	8.7311	6.39665	4.0456	**30**	29.239	30
24	4	3.2697	3.3064	2.5853	2.26775	**10**	10.888	10
120	4	5.5187	6.03515	4.73315	3.7837	**20**	19.840	20
311	4	9.3794	9.4273	7.93495	6.1031	**30**	31.278	30
24	8	3.81395	4.07665	3.1419	2.62495	**10**	13.217	10
69	8	6.13685	6.1534	5.2455	4.3246	**20**	21.189	20
175	8	9.1766	9.3283	7.932	6.78135	**30**	31.155	30
16	12	3.6754	3.79265	3.39145	2.96915	**10**	11.497	10
48	12	6.8696	6.8725	5.44085	4.9119	**20**	22.888	20
100	12	8.88765	8.89565	7.7497	6.72955	**30**	30.228	30
12	16	3.8322	3.79655	2.95985	3.15915	**10**	9.926	10
36	16	6.82745	7.197	5.9492	5.69395	**20**	22.367	20
77	16	9.203	9.132	7.85485	7.19205	**30**	30.242	30

## Data Availability

The initial experimental datasets used in this study were provided personally to the corresponding author and are not available.
